# Co-culture pellet of human Wharton’s jelly mesenchymal stem cells and rat costal chondrocytes as a candidate for articular cartilage regeneration: in vitro and in vivo study

**DOI:** 10.1186/s13287-022-03094-6

**Published:** 2022-07-30

**Authors:** Kaiwen Zheng, Yiyang Ma, Cheng Chiu, Yidan Pang, Junjie Gao, Changqing Zhang, Dajiang Du

**Affiliations:** Department of Orthopedic Surgery, Shanghai Jiao Tong University Affiliated Sixth People’s Hospital, 600 Yishan Road, Shanghai, 200233 China

**Keywords:** Human Wharton’s jelly-derived mesenchymal stem cell, Costal chondrocyte, Co-culture system, Chondrogenesis, Osteochondral defect, Cartilage regeneration

## Abstract

**Background:**

Seeding cells are key factors in cell-based cartilage tissue regeneration. Monoculture of either chondrocyte or mesenchymal stem cells has several limitations. In recent years, co-culture strategies have provided potential solutions. In this study, directly co-cultured rat costal chondrocytes (CCs) and human Wharton’s jelly mesenchymal stem (hWJMSCs) cells were evaluated as a candidate to regenerate articular cartilage.

**Methods:**

Rat CCs are directly co-cultured with hWJMSCs in a pellet model at different ratios (3:1, 1:1, 1:3) for 21 days. The monoculture pellets were used as controls. RT-qPCR, biochemical assays, histological staining and evaluations were performed to analyze the chondrogenic differentiation of each group. The 1:1 ratio co-culture pellet group together with monoculture controls were implanted into the osteochondral defects made on the femoral grooves of the rats for 4, 8, 12 weeks. Then, macroscopic and histological evaluations were performed.

**Results:**

Compared to rat CCs pellet group, 3:1 and 1:1 ratio group demonstrated similar extracellular matrix production but less hypertrophy intendency. Immunochemistry staining found the consistent results. RT-PCR analysis indicated that chondrogenesis was promoted in co-cultured rat CCs, while expressions of hypertrophic genes were inhibited. However, hWJMSCs showed only slightly improved in chondrogenesis but not significantly different in hypertrophic expressions. In vivo experiments showed that all the pellets filled the defects but co-culture pellets demonstrated reduced hypertrophy, better surrounding cartilage integration and appropriate subchondral bone remodeling.

**Conclusion:**

Co-culture of rat CCs and hWJMSCs demonstrated stable chondrogenic phenotype and decreased hypertrophic intendency in both vitro and vivo. These results suggest this co-culture combination as a promising candidate in articular cartilage regeneration.

## Introduction

Articular cartilage injury has a variety of causes and leads to pain and dysfunction of joints and even degenerative joint diseases such as osteoarthritis [1]. Owing to the avascular structure and extracellular matrix (ECM) which hinders the migration of cells, articular cartilage exhibits minimal self-regeneration capacity [2]. In recent decades, cartilage tissue regeneration strategies such as autologous chondrocyte implantation have attracted increasing attention [3]. In cell-based cartilage regeneration strategies, cell resources can be majorly divided into two groups: chondrocytes and mesenchymal stem cells (MSCs).

Chondrocytes isolated from articular cartilage are limited in number and tend to dedifferentiate during in vitro expansion [4, 5]. Contrastingly, due to their high proliferation, availability and chondrogenic potential, MSCs have been widely studied as cell sources for cartilage regeneration. However, MSCs usually undergo an unstable chondrogenic process characterized by hypertrophy and calcification [6–9].

Co-culture techniques of articular chondrocytes and MSCs have been investigated for cartilage tissue engineering in recent years and have demonstrated effective modulation of chondrocyte phenotype maintenance and MSC chondrogenesis promotion [10]. Direct co-culture systems provide a microenvironment for intercellular crosstalk between chondrocytes and MSCs, including direct cell–cell contact, cell–ECM contact and paracrine signaling [11]. In addition, MSCs partially replaced chondrocytes in the co-culture technique to reduce the number of chondrocytes required, thereby lowering the risk of chondrocyte dedifferentiation during expansion [12, 13]. Under co-culture conditions, chondrocytes expressed higher levels of ECM production as well as *COL2*, *SOX9*, and *ACAN* [14–17]. Overall, MSCs have been shown to exhibit a better chondrogenic and less hypertrophic phenotype [13, 18, 19].

However, articular chondrocytes have limitations in cartilage tissue engineering. Due to the limited number of available articular chondrocytes and potential donor site morbidity, non-articular chondrocytes such as costal chondrocytes (CCs) have been proposed as promising alternative sources [20]. Costal cartilage is the largest permanent hyaline cartilage storage in the mammalian body with several advantages over articular cartilage including low donor site morbidity, higher initial cell yield and proliferation rate, and better re-differentiation ability [21–24]. Therefore, it has been widely used in craniofacial microsomia, tracheal reconstruction and congenital tracheal stenosis [25]. Recent clinical trials using CC-derived pellets to restore cartilage defects in knees have also achieved satisfactory results [26, 27]. Therefore, we consider the costal chondrocytes as a possible alternative to articular chondrocytes.

Several different types of MSCs have been proposed as potential cell sources for cartilage repair, such as bone marrow-derived MSCs, adipose tissue-derived MSCs, synovial-derived MSCs and Wharton's jelly MSCs [28]. Human Wharton’s jelly mesenchymal stem cells (hWJMSCs) derived from the human umbilical cord have unique advantages including high proliferation rate, good freeze–thaw properties, multiple lineage differentiation potentials and immune privilege [29, 30]. Previous clinical trials have found that hWJMSCs can alleviate osteoarthritis and pain [31]. More importantly, the harvest and isolation of hWJMSCs from the umbilical cord is noninvasive without ethical controversy. Thus, it is regarded as an appealing cell source for articular cartilage regeneration.

As reported in previous studies, CCs have a stronger tendency for hypertrophy and ossification, which are considered important pathological changes in osteoarthritis cartilage [32–34]. The co-culture strategy mentioned above could be a potential solution for this phenomenon. Therefore, in this study, we chose a co-culture of rat CCs and hWJMSCs to study their capability for articular cartilage repair. These two cell types were directly co-cultured in a pellet model to explore the optimal co-culture proportion in vitro and their commitment to the co-culture environment via species-specific gene expression analysis. We also implanted co-cultured pellets in a rat articular cartilage defect model to validate their regeneration effects in vivo.

## Methods

All procedures using animals in this study were performed at animal experiment platform of Shanghai Model Organisms Center, Inc., in accordance with NIH Guidelines for the Care and Use of Laboratory Animals and were approved by Institutional Animal Care and Use Committee of Shanghai Model Organisms Center, Inc., (No. 2021-0023-06) and Animal Care and Use Committee of Shanghai Sixth People’s hospital (No. DWLL2022-0431).

### Characterization of hWJMSCs and isolation of chondrocytes

The hWJMSCs were generously given as a gift by Prof. Tao Ren from the Department of Respiratory Medicine. For surface marker identification, hWJMSCs of passage 5 were selected for flow cytometry analysis. Cells were suspended in phosphate-buffered saline (PBS), and a 100 μL sample was incubated with labeled mouse anti-human antibodies. Surface markers CD90, CD73, CD105, CD34, CD44, CD45 and HLA-DR were analyzed. Data were obtained from over 10,000 events per analysis.

The rat costal chondrocytes were obtained from 10 to 12 weeks old SD rat. Costal cartilage samples were minced to 1 mm^3^ and washed by PBS. The first step of digestion was carried out in 1.5 mg/mL type II collagenase in Dulbecco’s modified Eagle’s medium (DMEM) for 2 h at 37 °C, and the second step of digestion was with 0.75 mg/mL type II collagenase in DMEM overnight. After filtration with 70 μm sieves and centrifuging at 1500 rpm for 5 min, cells were collected and seeded onto a culture dish at a density of 1 × 10^4^ cells/cm^2^ in growth medium (α-MEM; 10% fetal bovine serum (FBS) and 1% penicillin/streptomycin). The medium was changed every 2 days. At 80–90% confluence, cells were digested with 0.25% trypsin/ethylenediaminetetraacetic acid (EDTA) and seeded onto new dishes at the constant density. Cells were all cultured at 37 °C with 5% CO_2_. Chondrocytes of passage 3 (P3) were chosen for the following study.

### Chondrogenesis in pellet culture

Following digesting and counting, cell suspensions containing 5 × 10^5^ rat costal chondrocytes (CC group) or hWJMSCs (SC group) or combinations of chondrocytes and hWJMSCs in different ratios (Table 1) were centrifuged at 1,500 rpm for 4 min to form pellets. All pellets were cultured in chondrogenic differentiation medium (DMEM, 2% FBS, 10 ng/mL TGFβ3 (PeproTech Inc., USA), 100 nM dexamethasone, 50 ug/mL ascorbic acid 2-phosphate, 1 mM sodium pyruvate, 40 ug/mL proline, 1% ITS (Gibco, USA), 1% penicillin/streptomycin) and incubated at 37 °C in a 5% CO_2_ incubator. The chondrogenic medium was changed twice a week until day 21.

**Table 1:** Co-culture ratio and cell number of different groups

Group	CC	3CC1SC	1CC1SC	1CC3SC	SC
Ratio	4:0	3:1	1:1	1:3	0:4
Rat costal chondrocyte (cells)	5 × 10^5^	3.75 × 10^5^	2.5 × 10^5^	1.25 × 10^5^	0
hWJMSC (cells)	0	1.25 × 10^5^	2.5 × 10^5^	3.75 × 10^5^	5 × 10^5^

### Biochemical analysis

To analyze glycosaminoglycan (GAG) synthesis, total GAG and DNA were measured. Cell pellets were digested in papain buffer (5 mM L-cysteine, 200 µg/mL papain, 0.1 M sodium acetate) for 18 h at 65 °C and centrifuged for 5 min at 6000 rpm. Subsequently, samples were assayed by dimethylmethylene blue assay following previous protocol [35]. GAG levels were determined by absorbances measured at 525 nm and standardized with chondroitin sulfate (Targetmol, USA). The DNA of pellets was extracted using an Animal Tissues DNA Extraction Kit (Solarbio, China) following the manufacturer’s instructions and measured with Nanodrop ONE (Thermo Scientific, USA). GAG synthesis was presented as GAG content normalized by DNA content.

### Quantitative real-time polymerase chain reaction (RT-qPCR)

The total RNA was extracted from pellets with Tissue RNA Purification Kit PLUS (EZBioscience, USA), and complementary DNA was prepared by using 4 × EZscript Reverse Transcription Mix II (EZBioscience, USA) according to the manufacturer's instructions. RT-qPCR was performed in a volume of 10 μL. Complementary DNA was amplified using specific primers and SYBR Green Master Mix with QuantStudio™ 7 Flex real-time PCR System (Thermo Fisher Scientific, USA). The amplification was performed under certain conditions: 5 min at 95 °C to activate, followed by 40 cycles, 15 s at 95 °C and 60 s at 60 °C. RT-qPCR was performed under standard conditions, and all experiments were performed in triplicate. The expression level of each gene was calculated using the 2^−(ΔΔCT)^ method with glyceraldehyde 3-phosphate dehydrogenase (GAPDH) as the reference gene. Primers were synthesized by Tsingke Biotechnology Co., China, and sequences are shown below (Table 2).

**Table 2:** Species-specific forward (F) and reverse (R) primers used for quantitative RT-PCR

Rat	*Gapdh*	NM_017008.4	F	CTGGAGAAACCTGCCAAGTATG
			R	GGTGGAAGAATGGGAGTTGCT
	*Sox9*	NM_080403.1	F	CACCAGCGTCAGTGAGGAAG
			R	GTCCAAACAGGCAGGGAGAT
	*Col2a1*	NM_012929.1	F	GGCCAGGATGCCCGAAAATTA
			R	ACCCCTCTCTCCCTTGTCAC
	*Acan*	XM_039101034.1	F	GGACAGAAGCCAGCACAGAG
			R	CTGCCAGTTGGGGCAGTTAT
	*Col10a1*	XM_001053056.8	F	ATGGCTTCACAAAGAGCGGA
			R	CCTACCCAAACGTGAGTCCC
	*Alpl*	NM_001127501.4	F	CCTACGCACCCTGTTCTGAG
			R	GGAAGTGAGGCAGGTAGCAA
	*Mmp13*	NM_133530.1	F	TCCATCCCGAGACCTCATGT
			R	GCAGCACTGAGCCTTTTCAC
Human	*GAPDH*	NM_001127501.4	F	GGAAGCTTGTCATCAATGGAAATC
			R	TGATGACCCTTTTGGCTCCC
	*SOX9*	NM_000346.4	F	ACAACCCGTCTACACACAGC
			R	CAAGTGGGTAATGCGCTTGG
	*COL2A1*	NM_001844.5	F	ACGTGAAAGACTGCCTCAGC
			R	CTGTCCCTTTGGTCCTGGTT
	*ACAN*	NM_001135.3	F	AAGGGCGAGTGGAATGATGT
			R	CGTTTGTAGGTGGTGGCTGTG
	*COL10A1*	NM_000493.4	F	CCAGCACGCAGAATCCATCTGA
			R	CCTGTGGGCATTTGGTATCGT
	*ALPL*	NM_000478.6	F	CCTGAGCGTCCTGTTCTGAG
			R	TCTTGGGTCCCCTTTCTTGC
	*MMP13*	NM_002427.4	F	TGAGCTGGACTCATTGTCGG
			R	GAGCCTCTCAGTCATGGAGC

### Animal experiments

To further investigate the effectiveness of pellets in cartilage defect repair, pellets after a 3-week induction were implanted into the cartilage defect in a rat model. A total of 24 twelve-week-old male SD rats were randomly divided into four groups (*n* = 4 knees per group at each time point): Blank group (defect only, untreated), CC group (rat costal chondrocyte pellets implanted), co-culture group (pellets of costal chondrocyte: stem cell, 50:50) and SC (hWJMSC pellets implanted). After general anesthesia and sterilizing, the rats' knee joints were opened with a medial parapatellar longitudinal skin incision. After the patella was dislocated laterally, a 1.5-mm-diameter osteochondral defect was made. Each group was treated accordingly. The joint capsule and skin were then closed. The rats were allowed to move freely in the cage after the operation.

### Macroscopic evaluation

For pellets, the gross morphology was examined after 21 days of culture in vitro. The size of the pellets was accurately measured using Image J software. At 4, 8 and 12 weeks post-surgery, the rats (8 rats at each time point) were killed by overdose intraperitoneal injection of pentobarbital sodium. The defect sites on femur grooves were imaged for quantitative evaluation by the International Cartilage Repair Society (ICRS) macroscopic score [36].

### Histology and immunohistochemistry

Pellets and samples from each group were fixed in 4% paraformaldehyde, decalcified (femur samples only) in 10% EDTA, embedded in paraffin, cut into 6-μm slices and mounted onto adhesive slides. Sections were stained with hematoxylin–eosin (H–E) and Safranin-O staining and evaluated with ICRS Visual Histological Assessment Scale (ICRS-VHAS) and O’Driscoll score [37, 38]. To evaluate the production of collagen type II and X histologically, immunohistochemical staining was performed in each group. Briefly, after deparaffinization, rehydration and antigen retrieval using Tris–EDTA, sections were incubated with rabbit antibodies against collagen type II (1:100, Affinity Biosciences, China) or collagen type X (1:50, Affinity Biosciences, China), followed by goat anti-rabbit secondary antibody conjugated with HRP (1:200, Affinity Biosciences, China). The area of the immunocomplex was visualized by chromogen 3,3′-diaminobenzidine (DAB, Beyotime, China) for 3 min. ImageJ software was used to analyze the integrated optical density and area to calculate the average of intensity (AOI) of images.

### Statistical analysis

All histological scores were evaluated independently by three blinded observers. The data are presented as the mean ± standard deviation. One-way analysis of variance (ANOVA) was used to determine the significant differences using Prism 8.0 software (GraphPad). A value of *p* < 0.05 was considered to indicate a significant difference.

## Results

### Cell characterization

**Fig. 1:**
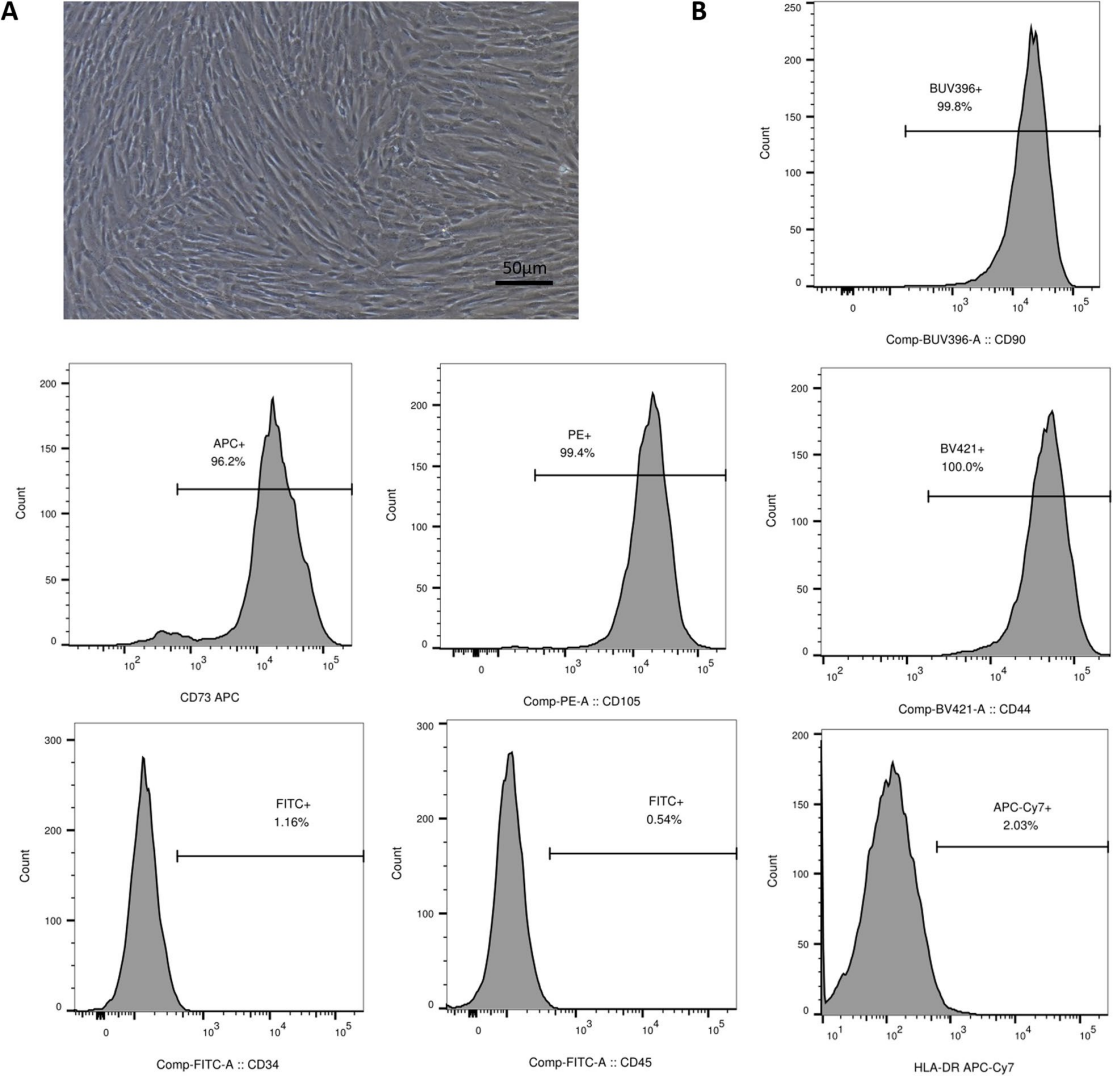
Cell morphology and surface marker confirmation of hWJMSCs. **A** Cultured hWJMSCs demonstrated typical spindle shape and a vortex distribution. **B** Flow cytometric analysis of surface markers including CD90, CD73, CD105, CD44, CD34, CD45 and HLA-DR

Cultured hWJMSCs demonstrated typical spindle shape and a vortex distribution (Fig. 1A). Flow cytometry analysis showed surface markers CD90, CD73, CD105 and CD44 were positive, while CD34, CD45 and HLA-DR were negative (Fig. 1B). These results met the definition of mesenchymal stem cells [39].

### Macroscopic and biochemistry evaluation

**Fig. 2:**
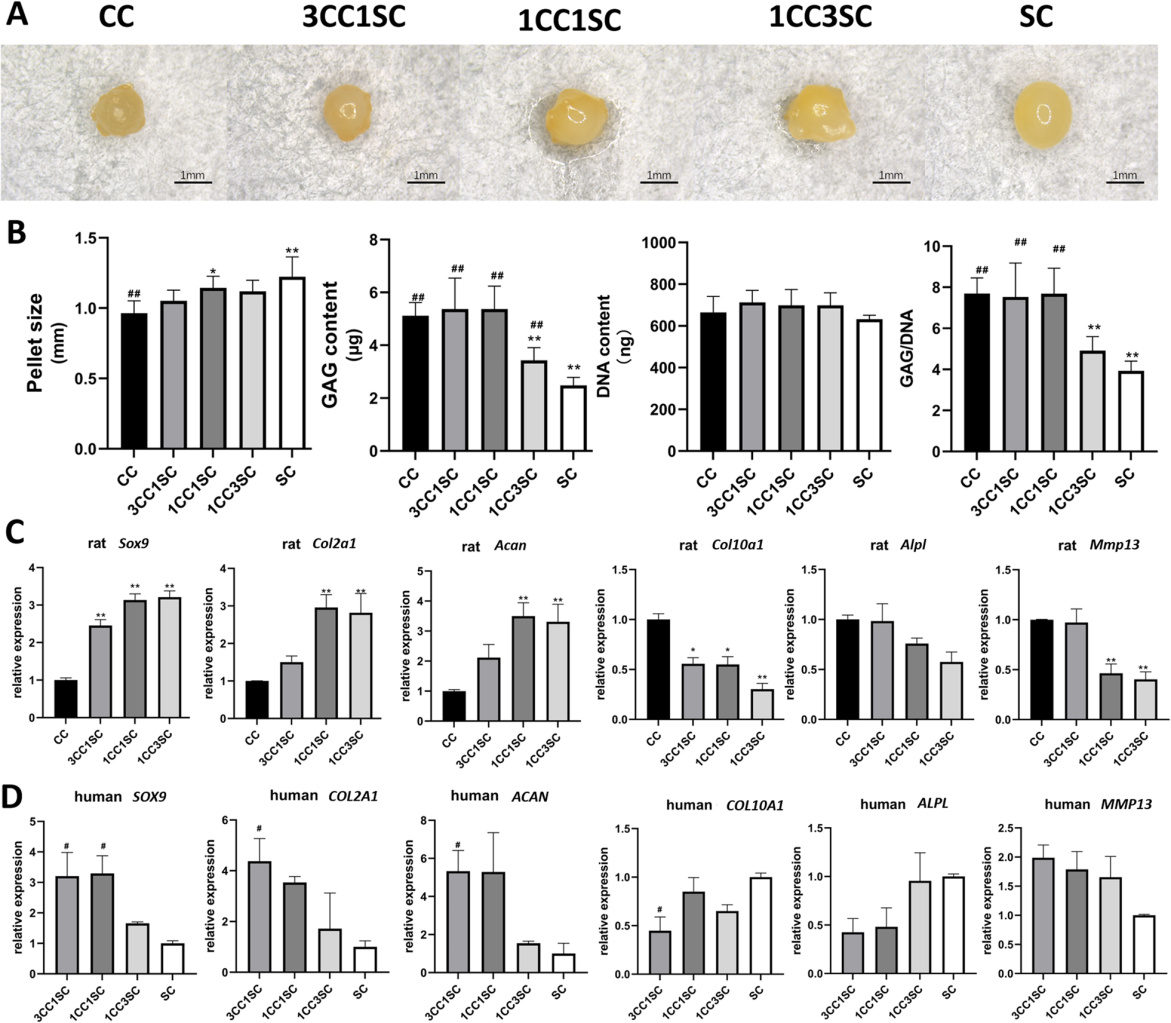
Morphology, biochemistry and RT-qPCR analysis of different groups of pellets. **A** Gross view of pellets in five groups. **B** Pellet size, GAG content, DNA content and GAG/DNA ratio analysis (*n* = 5). **C** Rat costal chondrocyte-specific gene expressions (*n* = 3). **D** hWJMSCs-specific gene expressions (*n* = 3). Significant difference symbols: *p < 0.05, **p < 0.01 compared to CCs group, #*p* < 0.05, ##*p* < 0.01 compared to hWJMSCs group

Five groups of pellets were harvested at day 21 further analysis. Macroscopic photographs indicated that pellets were spherical in shape and opaque appearance (Fig. 2A). Moreover, pellets with higher ratio of hWJMSCs presented slightly larger sizes (Fig. 2B). To evaluate cartilage matrix synthesis in different groups, GAG deposition and DNA content were also quantified (Fig. 2B). The data show that 3CC1SC and 1CC1SC groups demonstrated similar GAG content and GAG/DNA compared to CC group, while those of 1CC3SC and SC group were significantly lower (*p* < 0.01). The GAG/DNA ratio of the 1CC3SC group improved slightly in comparison with the SC group, but there was no significant difference (*p* = 0.10). There was no significant difference among these groups in DNA content.

### RT-qPCR analysis

To investigate the commitment of the two types of cells in the co-culture pellet, species-specific RT-qPCR was performed to investigate the chondrogenesis-related gene and hypertrophic-related gene expressions (Fig. 2C, D). Rat costal chondrocytes in 1CC1SC and 1CC3SC groups demonstrated significantly higher chondrogenesis gene expression (*p* < 0.01) as compared to the CC group, and the 3CC1SC group showed no different expressions of *Col2a1* and *Acan* (*p* = 0.76 and 0.22, respectively). As for hypertrophic genes, three co-culture groups showed decreased *Col10a1* expression (*p* < 0.05) and both 1CC1SC and 1CC3SC groups showed less *Mmp13* expression (*p* < 0.01). Despite the decreased expression of *Alpl* in co-culture groups, no statistical differences were found. In terms of gene expression changes of hWJMSCs, those in the 3CC1SC group showed a significant increase in *SOX9*, *COL2A1* and *ACAN* expression and a decrease in *COL10A1* expression (*p* < 0.05) compared to SC group and the 1CC1SC group showed a significant increase in *SOX9* expression (*p* < 0.05). Other results were not statistically different compared to the SC group.

### Histological evaluation of pellets

**Fig. 3:**
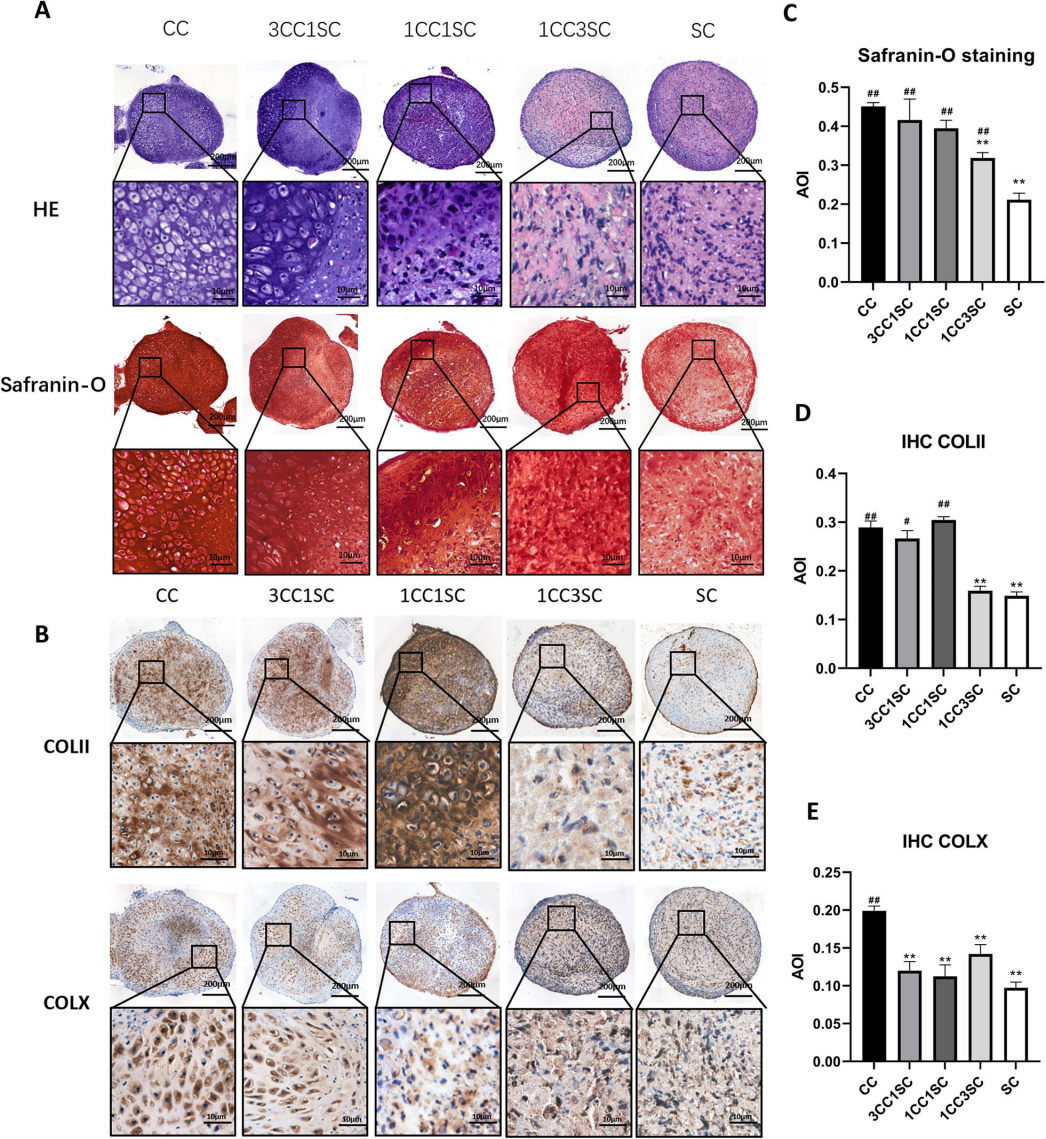
Histological and immunochemistry staining of pellets with semiquantitative analysis. **A** H–E and Safranin-O staining of pellets and partial enlargement in five groups. **B** Immunochemistry staining of COLII and COLX of pellets and partial enlargement in five groups. **C–E** Semiquantitative analysis of AOI of each staining (*n* = 4). Significant difference symbols: *p < 0.05, **p < 0.01 compared to CCs group, #*p* < 0.05, ##*p* < 0.01 compared to hWJMSCs group

We performed H–E and Safranin-O staining to validate the structure and GAG deposition and distribution (Fig. 3A). Cartilage-characteristic lacuna structure was observed in CC, 3CC1SC and 1CC1SC groups but not apparent in 1CC3SC and SC groups. All groups were positive in Safranin-O staining. And semiquantitative analysis indicated the Safranin-O staining was similar between CC and 3CC1SC, 1CCSC groups and significantly weaker in 1CC3SC and SC groups (*p* < 0.01) (Fig. 3C). In line with Safranin-O staining, immunochemistry staining of collagen type II showed similar positive staining distribution and semiquantitative results (Fig. 3B, D). As for immunochemistry staining of collagen type X, all groups showed significantly less deposition compared to the CC group (*p* < 0.01) but not statistically different compared to the SC group (Fig. 3B, E).

### Macroscopic evaluations of in vivo samples

**Fig. 4:**
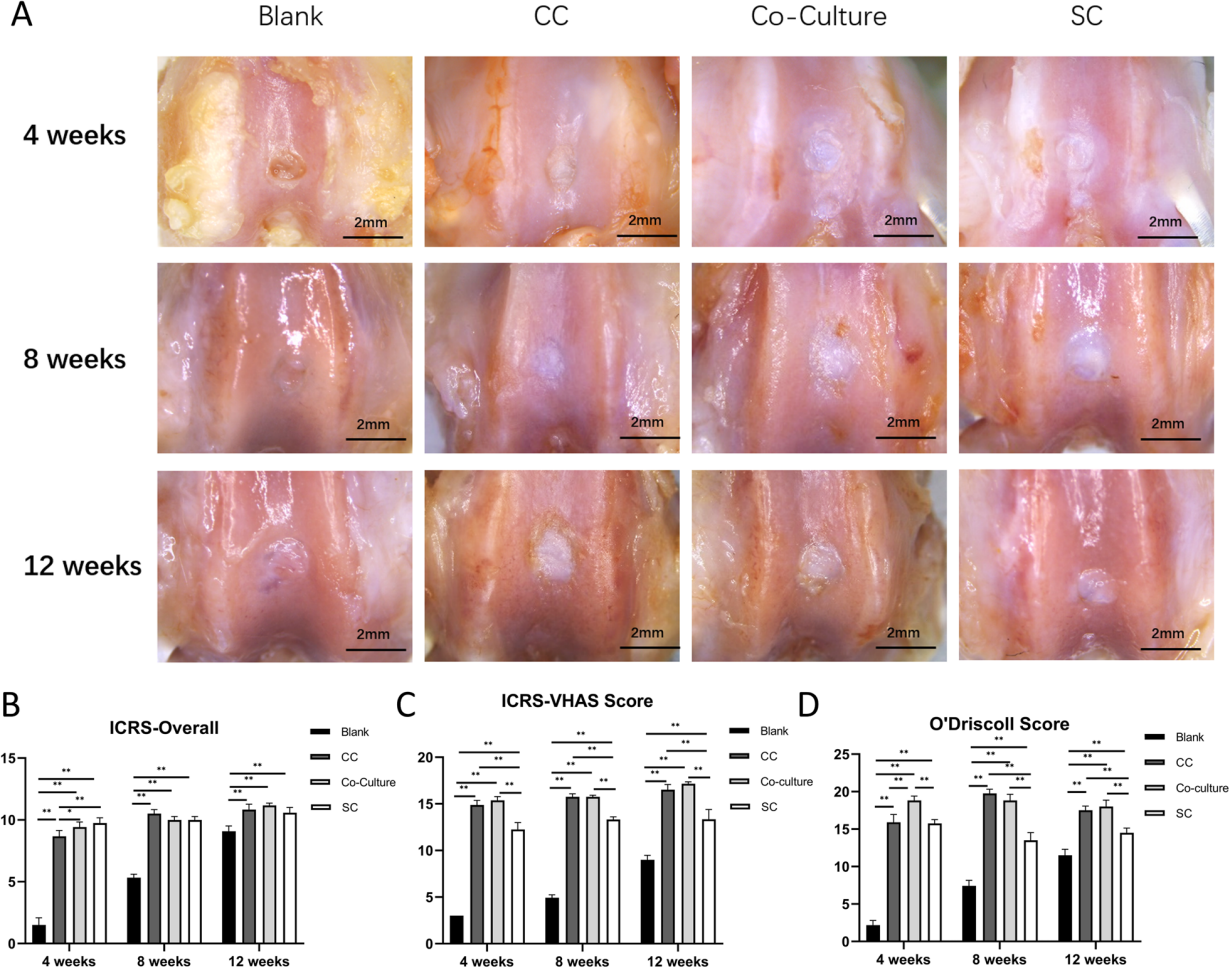
Morphology of in vivo specimens and evaluation scales. **A** Gross view of specimens in four groups at 4, 8, 12 weeks. **B**–**D** ICRS overall, ICRS-VHAS and O’Driscoll score of four groups at 4, 8, 12 weeks (*n* = 4). Significant difference symbols: *p < 0.05, **p < 0.01

Considering all these results above, we chose 1CC1SC as the optimal ratio for the co-culture group and further investigated the tissue repair effect in a rat osteochondral defect model. No death, infections or rejections of animals were observed. The blank groups showed obvious defects at 4 and 8 weeks and slightly concaved surfaces at 12 weeks. All pellet-grafted groups maintained good restoration in the defect sites. Their defect areas presented white and smooth surface and good integration with surrounding tissue except some samples of the CC group which revealed cracks or fissures near the defect area and concaves in the surface. All samples in the co-culture and SC group showed complete filling of defect sites (Fig. 4A). ICRS overall macroscopic scores were evaluated from aspects of macroscopic appearance, integration to the border zone and degree of defect repair (Fig. 4B). All the groups scored significantly at each time point with respect to blank group (*p* < 0.01). Co-culture pellet and SC pellet group score higher than CC group at the time of 4 weeks (*p* < 0.05 and 0.01, respectively) due to some irregularity of articular surface observed in CC group. But at the later time points, there are no statistical differences between the three pellet-grafted groups.

### Histological evaluation of in vivo specimens

We performed H–E and Safranin-O/Fast Green staining for the histological assessment and scored the sample by the standards of ICRS-VHAS and O’Driscoll scores.

**Fig. 5:**
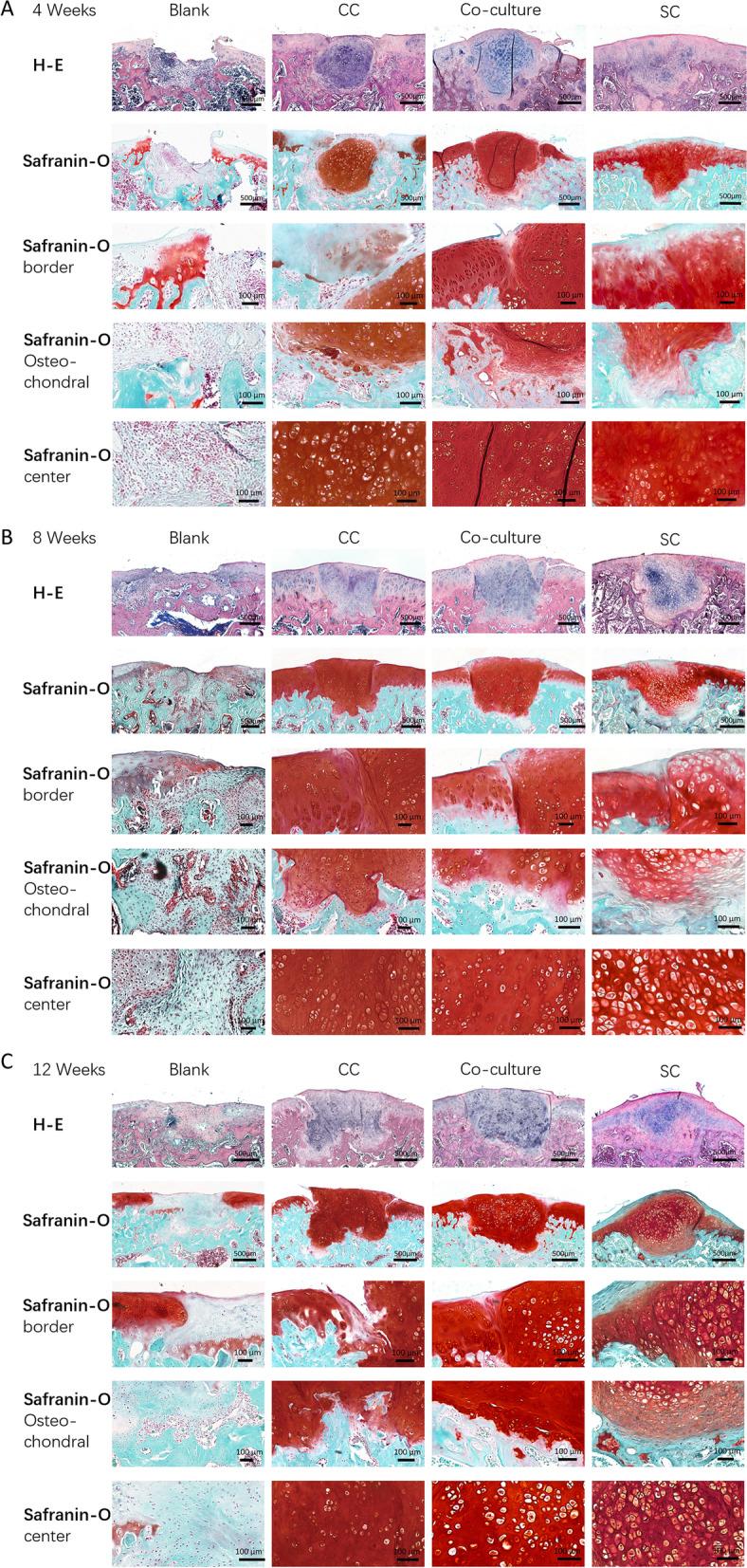
H–E and Safranin-O/Fast Green staining of specimens. **A** H–E and Safranin-O/Fast Green staining of four groups at 4 weeks. **B** H–E and Safranin-O/Fast Green staining of four groups at 8 weeks. **C** H–E and Safranin-O staining/Fast Green of four groups at 12 weeks. Border: enlargement of area between implanted pellet and surrounding cartilage, osteochondral: enlargement of interface between implanted pellet and subchondral bone, center: enlargement of pellet center

At 4 weeks, defect sites of the blank group are partially filled with irregular fibrous-like tissue without positive Safranin-O staining. All the grafted pellets showed intense Safranin-O staining indicating no signs of degradation. In addition, pellets of CC and co-culture group showed obvious cartilage tissue-like lacuna in the pellet after implantation. Some degeneration of surrounding cartilage was observed in the CC group with less strong integration between pellet and native cartilage but not observed in the other two groups. At this time point, the graft–host boundary in the subchondral bone area was unclear in all groups indicating the progress of subchondral bone remodeling (Fig. 5A).

At 8 weeks, defect areas are still filled with irregular fibrous tissue in the blank group. Pellets in all implantation groups showed better integration with surrounding tissue. Pellets of CC and co-culture group showed strong Safranin-O staining with lacuna structure. Pellet of SC group showed decreased Safranin-O staining and looser, hypertrophic-like matrix structure. These findings suggest that the matrix may experience degeneration at this time point. The subchondral bone areas of CC and co-culture group were more distinguishable with transition area between bone and pellet compared to previous time point, while those of SC groups remain unclear (Fig. 5B).

At 12 weeks, defect site of the blank group was almost the same level as native tissue. However, the content inside was hyaline-fibrous-like tissue with weak Safranin-O staining. Only the pellets of the CC group showed worse integration with the surrounding cartilage compared to 8 weeks suggesting the deficient durability of the CC pellets. Pellets of the SC group still showed loose and hypertrophic-like matrix structure and unclear boundary of subchondral bone area presenting delayed remodeling of subchondral bone. The subchondral area was improved in both the CC and co-culture pellet groups. The latter even demonstrated nearly the same structure as those of normal cartilage (Fig. 5C).

ICRS-VHAS scores of all the pellet-grafted groups were significantly higher compared to the blank group at all time points (*p* < 0.01). Those scores of CC and co-culture groups were significantly higher than the SC group at all time points (*p* < 0.01) (Fig. 4C). O’Driscoll scores showed results in accordance with ICRS-VHAS scores (Fig. 4D).

### Immunochemistry evaluation of in vivo specimens

**Fig. 6:**
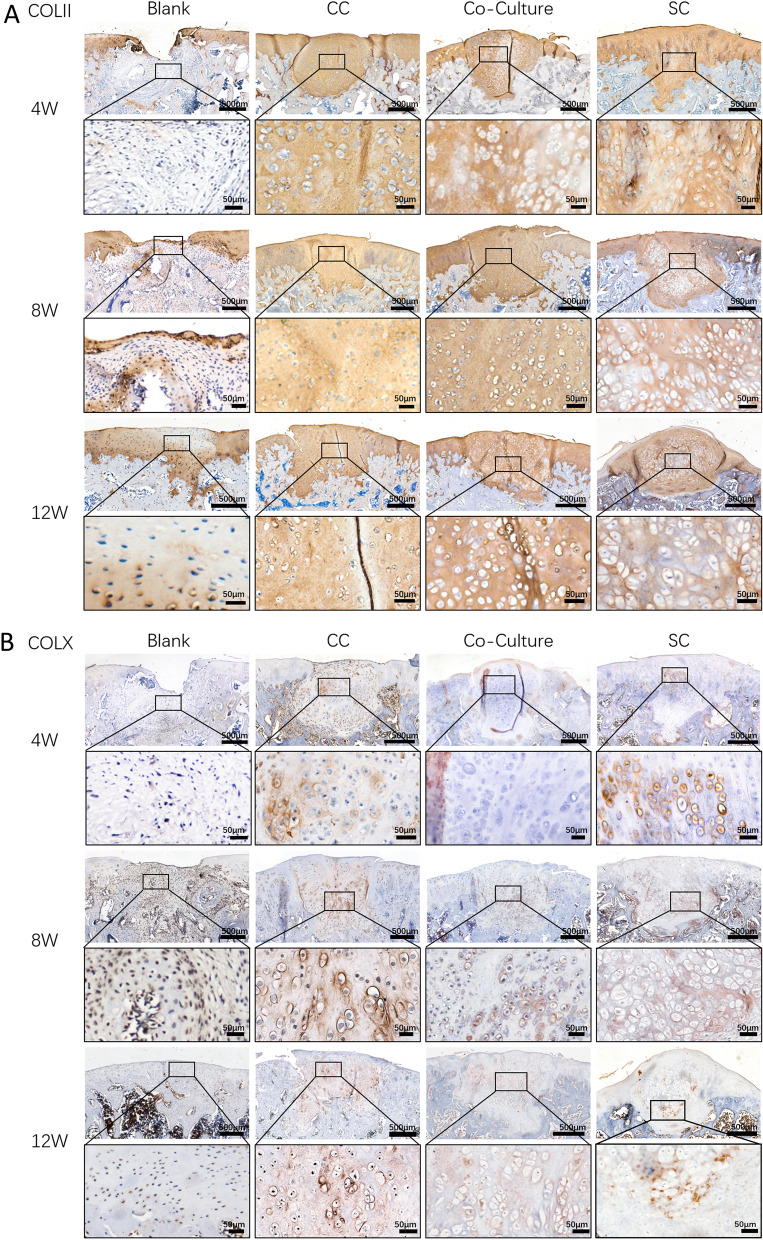
Immunochemistry staining of COLII and COLX of specimens. **A** Immunochemistry staining of COLII of pellet and partial enlargement. **B** Immunochemistry staining of COLX of pellet and partial enlargement

Immunochemistry staining of collagen II showed absence in defects at 4 and 8 weeks, while partial positive staining at 12 weeks in the blank group. For all the pellet implantation groups, collagen II was distributed uniformly in the defect area similar to the surrounding native cartilage. Only a slight staining decrease observed in the SC pellet group at 8 and 12 weeks due to the looser matrix. These findings suggest the pellets remained hyaline cartilage character up to 12 weeks in vivo. Collagen X immunochemistry staining demonstrated uniform positive staining area within the pellet and deeper staining density with post-implantation time in the CC pellet implantation group (Fig. 6A). The positive collagen X staining was also noticed in co-culture and SC pellet groups but with much weaker density and smaller area compared to the CC group. These results were in line with previous in vitro study that hypertrophic differentiation was mitigated in the co-culture group (Fig. 6B).

## Discussion

Both chondrocytes and MSCs have been studied as potential sources of cartilage regeneration. However, all exhibited unstable phenotypes under the monoculture conditions [13]. The co-culture system of chondrocytes and MSCs provides a promising solution to this phenomenon in cartilage tissue engineering, as it can reduce the number of chondrocytes required and promote ECM production [5, 13, 40]. In addition, previous studies revealed that direct co-culture systems showed better efficiency in the inter-cell synergistic effect than indirect ones [15, 41, 42]. Reasonable explanations may include cell–cell contact, autocrine and paracrine signaling and signal exchange through gap junctions [5, 12, 43, 44]. Therefore, we adopted a direct pellet co-culture model for this study. In terms of candidate seeding cells, we have noticed that in recent years, CCs have been regarded as a promising alternative for articular chondrocytes. Compared to that seen for articular chondrocytes, CCs have higher initial cell yield and proliferation rate, and higher *COL2A1* and *ACAN* expression after re-differentiation [22–24]. However, unlike articular chondrocytes, CCs tend to undergo hypertrophy and ossification after re-differentiation, which is unfavorable for articular cartilage regeneration [32–34]. Therefore, CCs have been evaluated as a suitable heterotopic cell source for articular cartilage engineering. Both articulate chondrocytes and CCs develop from the somites in the embryo and have similar abilities to produce a new cartilaginous matrix [24, 45]. Furthermore, CC applications in clinical treatments have been approved [25, 46, 47]. In order to improve chondrocyte phenotype of CCs, owing to the advantages such as high proliferation rate, multiple lineage differentiation potential, immune privilege and noninvasive isolation, hWJMSCs were chosen in the direct co-culture system in this study.

Different seeding cell combinations had different optimal mixing ratios [40]. Therefore, in the in vitro study, we compared different co-cultured pellets with chondrocyte–stem cell ratios (3:1, 1:1 and 1:3) with their monoculture groups in vitro. First, we evaluated the GAG content and GAG/DNA ratio and found that the 3:1 and 1:1 pellet co-culture groups had similar synthesis activities, while those of the 1:3 and stem cell monoculture groups were lower. Pellets of hWJMSCs seemed to produce less chondrogenic ECM than those with CCs. Semiquantitative analysis of Safranin-O and collagen II immunochemical staining showed consistent results. Previous studies have reported similar results for other co-culture combinations [42, 48–50]. A probable explanation for these findings is that chondrogenesis of chondrocytes rather than stem cells was predominant in the co-culture system. In contrast, chondrogenesis of chondrocytes in co-cultured groups was promoted by the trophic effects of stem cells [51–53]. Due to the trophic effects on chondrocytes, the matrix synthesis was similar in the 3:1 and 1:1 ratio groups when compared with that of the pure chondrocyte group in this study. Accordingly, there is a proposal that we should change the name of MSCs into medicinal signaling cells to focus on their ability to secrete trophic bioactive factors rather than their stemness [54].

To explore cell commitment in the co-culture system, the species-specific RT-qPCR was performed. The results showed that for rat CCs, the expression of chondrogenic-related genes (*Sox9*, *Col2a1*, and *Acan*) was enhanced and the expression of hypertrophic genes (*Col10a1*, *Alpl*, and *Mmp13*) was reduced in all co-culture groups. As for hWJMSCs, chondrogenic genes increased in 3:1 and 1:1 but not in 1:3. There was no significant difference in hypertrophic gene expression. These findings are consistent with those of our previous and some other studies, indicating that MSCs can stimulate chondrogenesis due to trophic effects on chondrocytes [50–53, 55–57]. However, the chondrogenic promotion and anti-hypertrophic effects of CCs on hWJMSCs were not significant. Possible explanations may be that (1) CCs have different inductive characteristics from articular chondrocytes, as they tend to promote osteogenic differentiation [58], and (2) although cultured in chondrogenic induction medium, hWJMSCs showed inferior chondrogenic differentiation to other types of MSCs, as has been previously reported [59–61]. However, all co-culture groups showed significantly lower collagen X production than the CC monoculture group, indicating the anti-hypertrophic effects of stem cells. These findings are in accordance with those of previous studies [5, 12, 40].

Based on the results of this in vitro study, we determined 1:1 as the optimal ratio of chondrocytes to stem cells according to ECM synthesis and hypertrophic tendency. This optimal ratio has also been determined in other chondrocyte and stem cell co-culture experiments [14, 62, 63]. Therefore, the 1:1 ratio pellet was further compared with CC and hWJMSC pellets for cartilage defect repair in vivo. In this study, all groups of pellets filled the defect sites and remained in situ for up to 12 weeks. Furthermore, histological and immunochemistry evaluation showed that the hyaline cartilage was positive for Safranin-O and collagen II staining, which was superior to that of the blank group at all time points. In addition, we noticed that pellets of hWJMSCs tended to demonstrate degenerate phenotype with weaker staining and decreased matrix at 8 weeks. This phenomenon could be attributed to chondrogenic induction of hWJMSC pellets before implantation because pre-differentiated MSCs are reported to result in poorer defect filling with fibrous-like cartilage and less collagen type II staining in vivo than undifferentiated MSCs [64–66]. We speculate that prolonged chondrogenic pre-differentiation may hinder the formation of cartilaginous matrix in situ and that the degree of successful chondrogenesis of MSCs in vitro does not guarantee superior cartilage repair in vivo [67, 68]. Meanwhile, pellets of CCs showed a sufficient cartilage phenotype but also hypertrophic differentiation after 4 weeks as seen in collagen X staining. As CCs experience hypertrophy and ossification spontaneously in the human body, they tend to develop hypertrophy after implantation [32, 33, 69]. Co-cultured pellets showed delayed and mitigated hypertrophy, which correlates well with in vitro study.

Some studies have demonstrated that poor integration between the repair or graft tissue and the surrounding host cartilage can lead to poor or failed tissue repair [70, 71]. For the in vivo part of this study, at 12 weeks post-implantation, the CCs group demonstrated dysconnectivity with native cartilage. In contrast, both the co-culture and MSC pellet groups showed satisfactory integration. We speculate that hWJMSCs have contributed to these connections because MSCs, whether pre-differentiated or not, have presented a favorable ability to bind surrounding cartilage in other studies [72, 73]. This may be related to N-cadherin-induced gap junction formation and increased cell adhesion of MSCs [74, 75]. In terms of osteochondral interface remodeling, we found that the bottom part of the pellets in the SC group showed less Safranin-O and some Fast Green staining after 8 weeks, indicating delayed subchondral bone remodeling. Considering the osteogenic differentiation potential of MSCs, these cells were likely to experience endochondral ossification. However, the mechanism of this phenomenon remains unclear and requires future investigation.

This study has some limitations. The related mechanisms of the signaling pathways and paracrine factors involved in co-culture and improved integration in co-culture groups have not been clarified. For delayed subchondral remodeling, 12 weeks may not be sufficient to observe the final development. In the future, more studies are needed to illustrate these relative mechanisms.

## Conclusion

Co-culture strategies using chondrocytes and MSCs demonstrated their advantages. In this study, CCs are chosen because of its abundance in storage, low donor site morbidity and good capacity of chondrogenic matrix synthesis. WJMSCs demonstrated trophic effects on phenotype maintenance and mitigated hypertrophy to costal chondrocytes, although chondrogenic contributions of themselves were not significant in this study. In vivo study confirmed the chondrogenic phenotype of co-culture pellets in the defect sites up to 12 weeks with reduced hypertrophy, better surrounding cartilage integration, and appropriate subchondral bone remodeling. This study suggests this co-culture combination as a promising candidate in articular cartilage regeneration.

## Data Availability

The datasets used and/or analyzed during the current study are available from the corresponding author on reasonable request.

## Abbreviations

ANOVA: Analysis of variance
AOI: Average of intensity
CCs: Costal chondrocytes
DAB: 3,3′-Diaminobenzidine
DMEM: Dulbecco’s modified Eagle’s medium
ECM: Extracellular matrix
EDTA: Ethylenediaminetetraacetic acid
FBS: Fetal bovine serum
GAG: Glycosaminoglycan
GAPDH: Glyceraldehyde 3-phosphate dehydrogenase
H–E: Hematoxylin–eosin
hWJMSCs: Human umbilical cord Wharton’s Jelly mesenchymal stem cells
ICRS: International Cartilage Repair Society
ICRS-VHAS: International Cartilage Repair Society Visual Histological Assessment Scale
MSCs: Mesenchymal stem cells
PBS: Phosphate-buffered solution
RT-qPCR: Quantitative real-time polymerase chain reaction
